# Dormancy-Related Seed Positional Effect in Two Populations of an Annual Grass from Locations of Contrasting Aridity

**DOI:** 10.1371/journal.pone.0093061

**Published:** 2014-03-27

**Authors:** Sergei Volis

**Affiliations:** Key Laboratory for Plant Diversity and Biogeography of East Asia, Kunming Institute of Botany, Chinese Academy of Sciences, Kunming, China; Ben-Gurion University of the Negev, Israel

## Abstract

In grasses, variation in seed size and dormancy often results from a seed's position within a dispersal unit. In this paper, I asked whether seed positional effect within a spikelet contributes to ecotypic differentiation between two populations of *Avena sterilis* having different species range position and associated aridity. I created experimental seed banks in which germination of seeds (florets) having different positions within a spikelet was examined over three years. In addition, two germination tests were conducted under controlled conditions. The two populations were found to have a short-living soil seed bank due to sequential germination of the florets. Although positional seed dormancy effect in *A. sterilis* does not appear to be a specific desert adaptation against unpredictability of rainfall events, this trait does contribute to ecotypic differentiation between desert and Mediterranean populations. Consistent with bet hedging buffering against rainfall unpredictability, germination fractions in the first year were higher in the Mediterranean than in the desert population, while seeds of the desert origin had stronger dormancy and more sequential germination of florets.

## Introduction

Intra-individual variation in seed size and/or morphology is often associated with variation in seed dormancy [reviewed in 1–2]. Variation in seed size (with a concomitant effect on dormancy) can take the form of seed heteromorphism, viz. production of different kinds of seeds by a single individual. This is a well known phenomenon in plants [reviewed in 3] but most examples of this phenomenon come from two families, Asteraceae and Chenopodiaceae, while seed heteromorphism and its ecological consequences in Poaceae are poorly described. Moreover, most known examples of Poaceae seed heteromorphism are cleistogamous vs. chasmogamous flower derived seeds [4].

However, in Poaceae family, variation in seed size and dormancy often results from a seed position within an inflorescence and within a dispersal unit [5]–[6]. Many environmental factors that affect seed dormancy (water availability, temperature, light intensity) act at different points during seed development. Because the sub-units of the seed system (e.g. spikelets within an inflorescence or grains within a spikelet) have different rates and order of development, the latter determine the order of sequential loss of dormancy by these subunits [7]–[8]. In contrast to Asteraceae and Chenopodiaceae, seeds having different position within an inflorescence and within a dispersal unit often differ in size but not shape and this kind of seed heteromorphism is overlooked in the literature.

Variation in caryopsis dormancy among spikelets within a panicle was reported for *Oryza* [9], *Poa* [10] and *Avena* with dormancy increasing from the panicle top to its bottom [5]. In cereals with spiked inflorescences, such as *Triticum*, dormancy increases basipetally [6].

In several genera with a dispersal unit containing several seeds, the first formed and therefore better developed (and usually, although not always, larger) seeds have lower dormancy. In *Triticum* the upper grain in the spikelet is usually better developed, larger and exhibits less dormancy than the lower grain [11]. In *Eremopyrum* (*E. distans*) the basal grains are largest, but as in *Triticum* more dormant than other grains [12]. However, in *Aegilops*, *Agrostis*, *Poa* and *Avena* the lower (basal) grain is formed first, has larger size and more rapid germination than the more proximal one [13]–[14]. Studies of genetically distinct lines of *A. fatua* showed that temperature and photoperiod interact with the genetic determinants of panicle morphology resulting in varying degree of dormancy of the caryopses within a panicle. High grain dormancy was associated with long grain development period, many small grains and high grain water content, while low dormancy lines had short grain development times and few, heavy grains with low water content [15]–[16].

Germ banking, i.e. bet-hedging against future environmental stochasticity through variation in time of emergence among genetically identical offspring [17] is one of the most important adaptations to unfavorable and unpredictable environment. An important advantage of a germ banking, such as diapause or dormancy, is in buffering against catastrophic extinctions that otherwise are unavoidable (e.g. population failure to reproduce for more than one year) [18]–[19]. Higher probability of extinction in a stochastic environment was shown for populations with shorter-lived propagules both theoretically [20]–[21] and in a case study [22].

A particular form of germ banking, seed dormancy, is a trait directly related to climatic conditions allowing spread of germination over several seasons, and seed banks are recognized for their importance in population dynamics of both perennials and annuals [23]–[24]. It is generally predicted that in variable and unpredictable environments, the optimal germination strategy is reflected in a positive correlation between germination fractions and the probability of experiencing a favorable (i.e. rainy) year [25]–[26]. Therefore, we may expect position-related seed dormancy to vary among conspecific populations, with seed bank be more important in the dynamics of populations occupying temporally more heterogeneous and unpredictable environments.

In this paper, I asked whether seed positional effect within a spikelet contributes to ecotypic differentiation between Mediterranean and desert plants of *Avena sterilis* L., a widespread in Israel annual grass. Deserts are renowned for generally low, highly fluctuating and unpredictable precipitation from one year to the next [27], while Mediterranean-climate regions exhibit a longer growing season, more predictable onset of the rainy season, higher and more reliable within-season supply of water [28]. My prediction was that germination fraction will be lower in the desert vs. Mediterranean population.

The objective of this study was to test for an effect of a seed's position within a spikelet on its germination during three consecutive years in plants having Mediterranean and desert origin. This testing involved several experiments conducted under both natural and controlled environmental conditions.

## Materials and Methods

Beit Guvrin National Park and Mitrani Department for Desert Ecology, Institute for Desert Research, Ben-Gurion University permitted usage of their land for the experiment, it did not involve any endangered or protected species and no vertebrates were involved.

### Study species

*Avena sterilis* L. is a predominantly selfing winter annual grass. In Israel, this species is one of the major components of annual vegetation in the mesic Mediterranean including open park-forests, maquis and hemicryptophytic/dwarf shrub formations, and also penetrates into favorable desert habitats (wadi beds and loessy depressions) [29].

In this species the dispersal unit is a spikelet that usually contains two or three, rarely four flowers, the primary floret being larger and better developed than the secondary or tertiary grains. The fourth floret is nearly always undeveloped and sterile (Volis, personal observations). The inflorescence is a panicle with basipetal maturation of spikelets (from the periphery to the center). Within the spikelets, however, florets mature acropetally, from the basal floret up, and degree of dormancy follows this order. Seedlings emerge in November-December, grow and mature through winter - early spring, produce seeds in April-May and senesce before summer. Seeds that do not germinate in the autumn following dispersal either die or enter the soil seed bank where they can remain dormant for several years [30].

### Study sites

One research site was established per Mediterranean and desert climatic zone in Israel with a permission from corresponding authorities. The Mediterranean population (M) is in Beit Guvrin National Park located in the Shefela Hills (latitude 31.599, longitude 34.897, elevation 300 m, average annual precipitation 400 mm). The area is a semi-steppe batha on rendzina soil with mosaic of shrub – semi-shrub cover (*Sarcopoterium spinosum*, *Calicotome villosa*, *Cistus salvifolius*) and dense stands of *A*. *sterilis,* among other grasses.

The desert population (D) is in a wadi in the Negev Desert (latitude 30.853, longitude 34.765, elevation 400 m, average annual precipitation 90 mm), fenced as an experimental area of the Mitrani Department for Desert Ecology, Ben-Gurion University. There is sparse desert vegetation on loess soil (dominated by shrubs and semi-shrubs including *Retama raetam*, *Thymelea hirsuta*, *Zygophyllum dumosum*, *Hammada scoparia*) with patchily distributed *A*. *sterilis* within the wadi.

The D site was found to be less predictable in annual rainfall amount than the M site (CV in annual rainfall over 60 years is 0.43 and 0.32 in D and M site, respectively).

### Soil seed bank experiments

Two soil seed bank experiments were conducted in 1998–2000 and 2004–2007.

**Figure 1:**
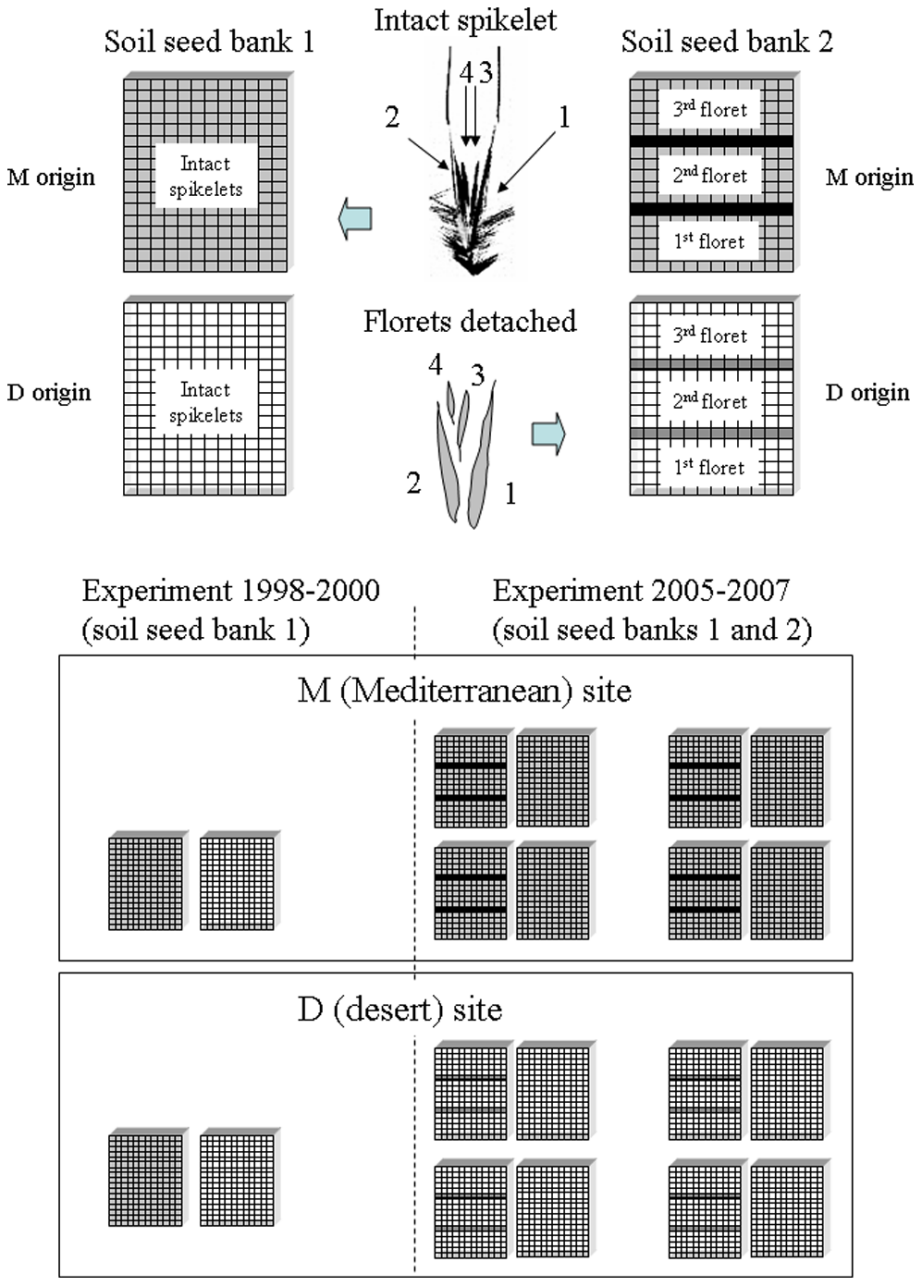
Floret position within *A. sterilis* spikelet (numbers denote floret order from the basal to the uppermost) and a scheme of two experimental soil seed banks (one with intact spikelets and another one with detached florets). In the experiment 1998–2000 trays with intact spikelets of M and D origin (grey and white color, respectively) were reciprocally introduced at the M and D sites. In the experiment 2005–2007 trays with both intact and detached florets of M and D origin were introduced at the location of origin.

**Figure 2:**
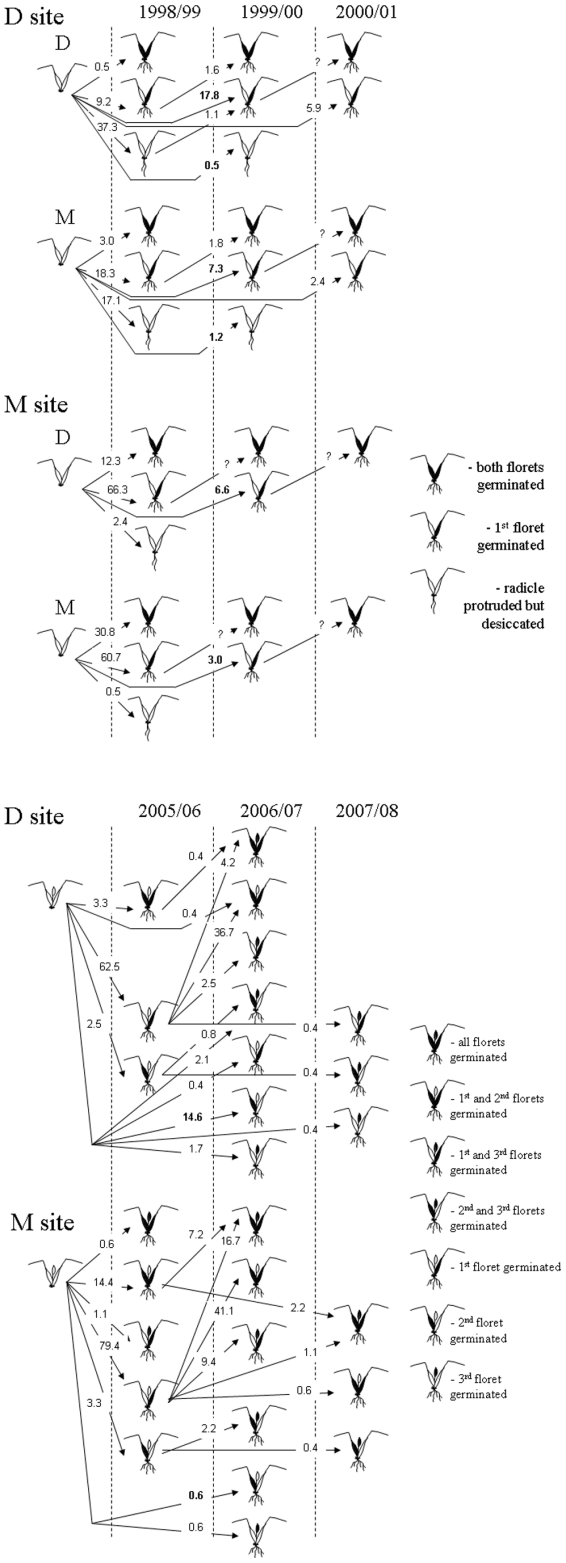
A scheme of spikelet germination over three consecutive years in two soil seed bank experiments conducted during 1998–2000 and 2005–2007. In the 1998–2000 experiment transitions (in % from the total 100% before burial) were recorded between the following spikelet states: before burial, desiccated after germination but able to develop upon wetting, one floret germinated, and two florets germinated. In the 2005–2007 experiment transitions (in %) were recorded between seven spikelet states that differed in the number and specific position of germinated florets within a spikelet. In the 1998–2000 experiment spikelets of two origins were reciprocally introduced at the two sites and in the 2005–2007 experiment only spikelets of local origin were introduced. Important transitions are in bold.

In 1997 seeds of *A. sterilis* were collected from randomly selected adult plants in proximity to the plots under observation. Fifteen spikelets from each population, M and D, were planted in the following season in a greenhouse at the Bergman Campus, Ben Gurion University in Beer Sheva (latitude 31.251, longitude 34.799) to obtain F1 seeds with maternal effects removed. Beer Sheva is located in a transition zone between the Mediterranean and desert climate in Israel (mean annual precipitation 202 mm). In 1998/99 220 spikelets collected from greenhouse-grown mother plants from each of the two populations were reciprocally buried at each transplant site. An intact spikelet was placed in a separated cell of a plastic tray filled with soil of the transplant environment and covered with fine metal net to prevent seed predation by ants, rodents and birds (soil seed bank 1 on Fig. 1). However, this net could not exclude seed predation by soil arthropods. The trays were buried to the surface level. Two months after the first effective rains (> 10 mm of rainfall) the trays were removed, brought to the laboratory and spikelets classified into the following groups: i) germinated (with the radicle protruded) but desiccated before coleoptile development and able to develop adventitious roots upon wetting (as determined in separate tests); ii) germinated and developed coleoptile or true leaves; iii) non-germinated. In 1999/2000 the non-germinated spikelets (excluding those with clear signs of predation which were excluded from the analysis) were buried again at the respective transplant sites and the procedure was repeated; this was repeated again in 2000/2001. There usually was from two to three weeks between exhumation and reburial. Germination percentages were calculated for each spikelet state in a given year from the total number of spikelets buried and Fig. 2 shows the percentages with a transition link to the spikelet state in a previous year.

In 2003 seed collecting and propagation was repeated as above. Equal numbers of spikelets of corresponding origins were used to create two spikelet pools, M and D. These pools provided spikelets for two types of experimental soil seed banks. The soil seed bank 1 was similar to one used in the experiment conducted during 1998–2000 (Fig. 1). In this soil seed bank, each of 221 intact spikelets of local origin was placed in a separate 1.5×1.5 cm cell of a plastic tray (17×13 cells). In seed bank type 2, the florets comprising each of 60 spikelets of local origin were separated, and each placed in a separate 1.5×1.5 cm cell of a plastic tray 17×12 cells as shown in Fig. 1. The position of 1^st^, 2^nd^ and 3^rd^ florets was fixed within a tray and separated by a row of cells with no seeds inside to ease subsequent identification and prevent effects on each other. The trays were again filled with the local soil, covered with metal net, and buried in November 2004 at the site of spikelet origin. Two trays, representing two types of the soil seed bank, were buried together at each of the randomly chosen four blocks within each site, M and D. In the next three years, the trays were visited yearly two months after the first effective rains (> 10 mm of rainfall) and germination event was recorded for each cell of the tray. One tray with separated florets buried at the M site was lost due to vandalism. In this experiment, I was able to compare directly each year germination fraction in intact and disintegrated spikelets and to see a possible effect of floret disintegration on germination. A disintegrated spikelet was considered germinated in a given year if at least one of its dissected florets germinated. And, because I knew each floret's precise position in a tray and its initial spikelet position, it was possible to trace a fate of each floret over three years and to analyze jointly the effects of floret position and a year on floret germination.

In the first soil seed bank experiment, germination counts were compared by the chi-square test with Yates correction for continuity. In the second experiment, in addition to the chi-square test, I used the Generalized Linear Model (GLZ) of Statistica [31]. I fitted a multinomial linear model with a logit link to germination of detached florests. The response variable included three categories (1^st^, 2^nd^ and 3^rd^ year of germination). It was not possible to include the block (viz. tray) effect nested within a site and therefore germination of detached florets was analyzed with the model including two fixed effects, population origin ( =  transplant site) and floret position within a spikelet. For intact spikelets, I used a binomial linear model with a logit link and year as a fixed effect because each of the three florets within a spikelet could germinate in any given year. As a result a spikelet could germinate up to three times during three years of observation. The response binary variable included two categories (whether a spikelet germinated or not), and independent variables were three fixed effects, population origin ( =  transplant site), floret position within a spikelet, and a year.

### Germination under controlled conditions experiments

Germination of florets from different positions in a spikelet was tested under controlled conditions in a greenhouse at the Bergman Campus, Ben Gurion University in Beer Sheva. Prior to experiments, fifteen accessions of each, M and D origin were grown in a greenhouse and their seeds were pooled. The 75 of each, first, second and third floret were sown in November 2010 in pots (bottom ∅ 12 cm, top ∅ 20 cm, height 12 cm) filled with the soil from the plant origin location and received water treatment simulating first rainy event of different quantity. The amount of water applied was equivalent to 5, 10, 15, 20, 25, 30 and 40 mm of rainfall. This experiment was supposed to quantify a relationship between the amount of water in the first rainy event and germination fraction, and identify the amount of rainfall that is a trigger for mass germination in *A. sterilis*. There were five replications for each treatment. In August 2011 the florets were dug out and classified as germinated or non-germinated. The non-germinated florets of five replications of particular origin and water treatment were pooled and sown again in pots and soil as above immediately after examination and stored outside in a roofed facility. The pots were irrigated in November 2011 as in the previous season and the whole procedure was repeated. The last irrigation was applied in November 2012 and in August 2013 the experiment was completed

An additional experiment was designed to test for any effect of soil type on seed germination in *A. sterilis*. In this experiment, 100 of each, first and second floret were sown in November 2010 in pots described above; filled with two soil types, rendzina and loess, corresponding to the native soil types of the M and D origins, respectively. The amount of water applied was equivalent to 15, 20, 25, 30 and 40 mm of rainfall. The timing of irrigation and all other details of the procedure were identical to the above greenhouse experiment.

Germination counts were compared by the chi-square test and Wilcoxon matched pairs test.

## Results

### Soil seed bank field experiment 1998-2000

A scheme of spikelet germination at the two sites over three consecutive years is depicted on Fig. 2. The spikelet germination significantly differed between the introduction sites in the first two years after introduction. In the first year, lower percentage of spikelets germinated (with either two florets germinated, one floret germinated or protruded radicle that subsequently desiccated) was observed at the D site (47.0%, 87/185 and 38.4%, 63/164, D and M, respectively), as compared with the M site (81.0%, 171/211 and 92.0%, 185/201, D and M, respectively) (χ^2^_1_ = 158, p<0.001), but spikelets of D and M origin did not differ in percent germination (χ^2^_1_ = 0.5, p>0.05). However, in the second year, the proportion of germinated spikelets that were dormant in a previous year was significantly higher at the D site (18.3%, 33/185 and 8.5%, 12/164, D and M, respectively) as compared with the M site (6.6%, 14/211 and 3.0%, 6/201, D and M, respectively) (χ^2^_1_ = 14.6, p<0.001), and D spikelets had higher germination proportion at both sites than M spikelets (χ^2^_1_ = 10.8, p<0.01). There was a higher percentage of desiccated spikelets at the D as compared with the M site, and some of the desiccated spikelets showed revival in the next season (Fig. 2). Germination over the first two years and especially over three years was more compromised at the D site compared to the M site (D site: 64.9%, 120/185 vs. 45.7%, 75/164, two years; 70.8%, 131/185 vs. 48.2%, 79/164, three years, χ^2^_1_ = 12.1 and 17.7, p<0.001, D and M origin, respectively; M site: 87.6%, 185/211 and 95.0%, 191/201; two and three years, χ^2^_1_ = 6.1, p<0.05, D and M origin, respectively).

In this experiment, spikelet viability was found not to exceed 3 years and order of floret germination to correspond to that of maturation, viz. the basal floret first, then the one above and so on. However, germination of third and forth florets were rare events (less than 1% of germinated spikelets) and were not accounted for. Germination of the second floret in spikelets with one floret germinated in a previous year was estimated in this experiment for the D site only, so it could not be compared across the two sites. In addition, the seedlings in this experiment were dug up at the age of two months, their aboveground parts were cut off and only the belowground parts containing non-germinated second floret were buried again. These shortcomings make it difficult to interpret observed germination pattern in the same spikelet as one occurring naturally. The latter required non-destructive (i.e., with no digging out and removal of aboveground tissue) floret germination censusing over time. This was done in the second experiment conducted during 2005–2007.

### Soil seed bank field experiment 2005–2007

**Figure 3:**
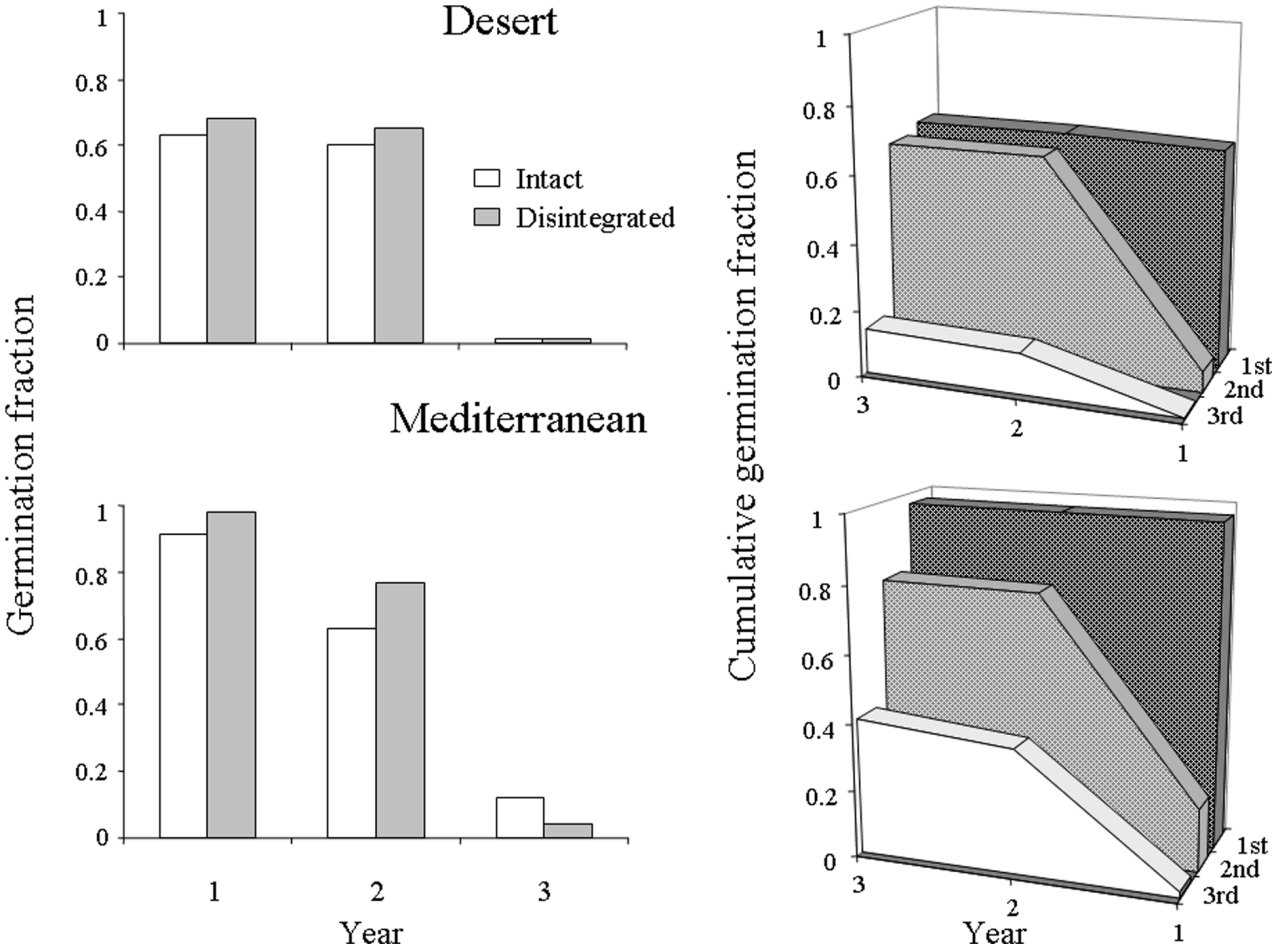
Left. Each year germination fraction in intact vs. disintegrated spikelets. A disintegrated spikelet was considered germinated in a given year if at least one of its dissected florets germinated. Right. Cumulative proportion of germinated florets of different position in a spikelet over three consecutive years.

The experiment of 2005–2007 allowed direct comparison of germination process in intact spikelets and detached florets. There was a good match at both sites between proportions of germinated spikelets over three years in intact and disintegrated spikelets (Fig. 3). Germination of florets was sequential, as in the experiment 1998–2000, but with a clearer pattern of spikelet position-dependent germination due to the better experimental design. Germination of the 1^st^ and 2^nd^ florets occurred during the first two consecutive years (1998–99 and 1999–00) with an exception of one 2^nd^ floret at M site that germinated in the third year. The 1^st^ (basal) florets germinated almost exclusively in the first year, and the 2^nd^ (one above the basal) florets germinated predominantly in the second year (Fig. 3). Most of the 3^rd^ florets also germinated in the second year, and only a few florets germinated in the preceding and the following years (Fig. 3).

Analysis of germination of intact spikelets during three years by GLZ with binomial model (two categories; germinated vs. non-germinated) revealed significant effect of year, site and their interaction (Wald Statistics 737, 145 and 126, p<0.001). The effects of site and site-by-year were significant because of higher germination at the M site in the first and third year (Fig. 3). However, in the analysis of floret germination during three years by GLZ with multinomial model (three categories; germinated in the 1^st^, 2^nd^ and 3^rd^ year) the effect of site was not significant (Wald Statistics 0.8, p>0.05), while effect of floret position within a spikelet was highly significant (Wald Statistics 138, p<0.001). The interaction between site and floret position was not significant (Wald Statistics 1.3, p>0.05).

At the M site, germination in the first year was higher than in the second (intact spikelets, 91%, 808/884 vs. 63%, 555/884, χ^2^_1_ = 203, p<0.001; disintegrated spikelets, 98%, 177/180 vs. 77%, 138/180, χ^2^_1_ = 36.7, p<0.001), while at the D site, it was similar (intact spikelets, 63%, 550/867 vs. 60%, 520/867, χ^2^_1_ = 2.0, p>0.05; disintegrated spikelets, 68%, 163/240 vs. 63%, 156/240 χ^2^_1_ = 0.3, p>0.05). Germination in the third year was much lower than in the first two years for both M and D, but still significantly different from zero for M (12%, 107/884 and 4%, 8/180, χ^2^_1_ = 99.2 and 5.9, p<0.001 and<0.05; intact and disintegrated spikelets, respectively). Germination of the D spikelets in the third year did not differ significantly from zero (χ^2^_1_ = 2.0 and 1.0, p>0.05; intact and disintegrated spikelets, respectively). The latter can be explained by a very dry winter in 2007 at the D site.

In the first and the second years, the germination fraction was higher at the M site as compared with the D site for intact spiklets (χ^2^_1_ = 25.0 and 67.5, p<0.001), but did not differ in the second year (χ^2^_1_ = 0.2, p>0.05). Similarly, for the disintegrated spikelets, the germination fraction was significantly higher at the M site than at the D site in the first year (χ^2^_1_ = 6.4, p<0.05), did not differ in the second year (χ^2^_1_ = 1.5, p>0.05) and was marginally significantly different in the third year (χ^2^_1_ =  2.8, p<0.10) (Fig. 3).

Germination fractions of the florets during three years at the M and D sites were either similar or higher at the M site, with one exception (Fig. 2). Germination of the 2^nd^ floret after one year in the soil seed bank was significantly higher at the D than the M site (14.6 vs. 0.6%, χ^2^_1_ = 20.6, p<0.001)**.**

The two seasons when the seeds were introduced differed in the first effective rain at the D site (10.8 vs. 14.4 mm, 1998/1999 and 2005/2006 seasons, respectively). The actual difference in germination conditions between these two seasons was larger because in 2005/2006, the rainy event of 14.4 mm was preceded by a rain in a previous day (4.0 mm) and followed by two days of rain (6.2 and 1.2 mm), which did not happen in the 1998/1999 season.

### Germination under controlled conditions

**Figure 4:**
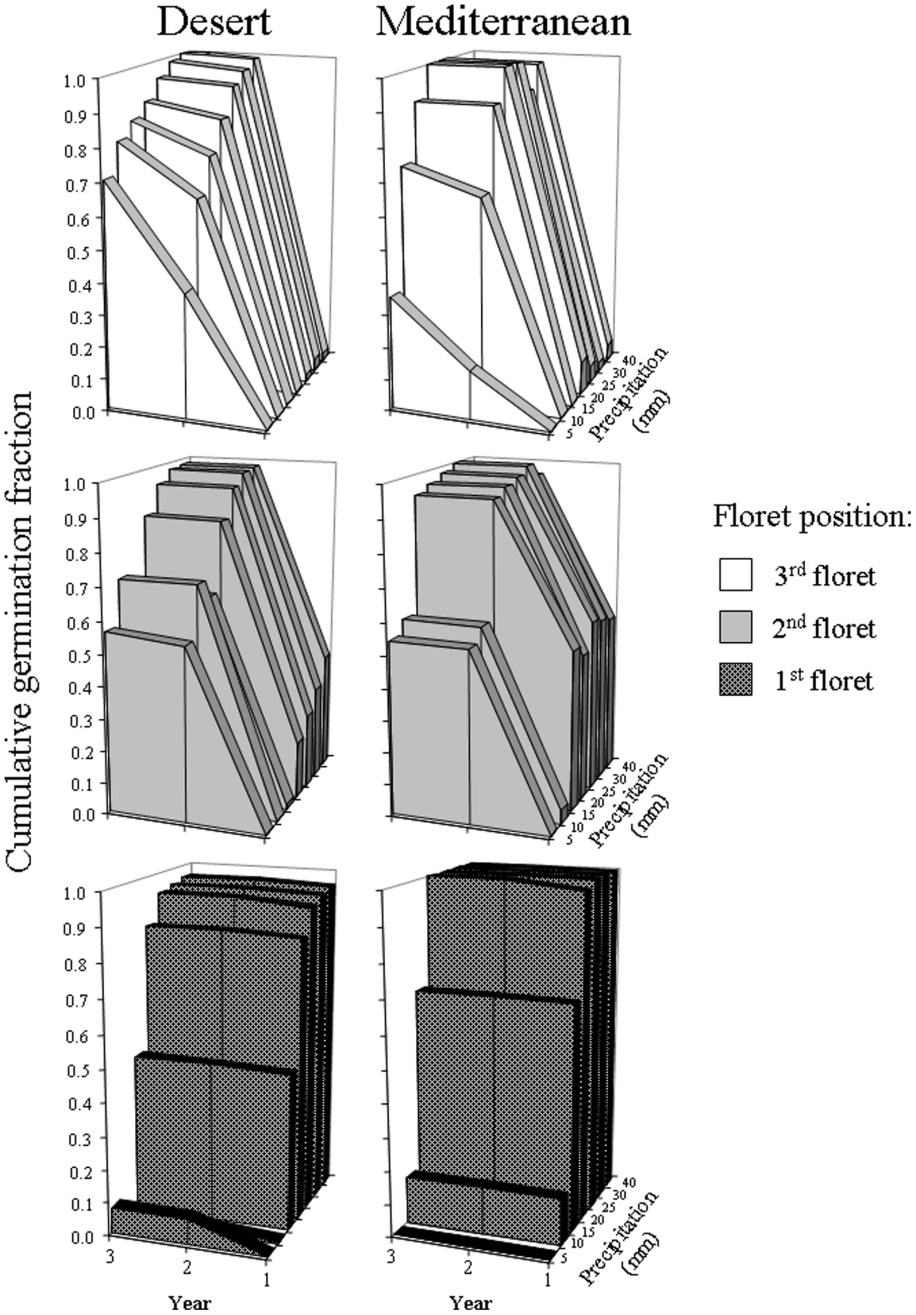
Cumulative germination fractions of the desert and Mediterranean florets of different position in a spikelet (1^st^, 2^nd^ and 3^rd^ position in a spikelet from the bottom to the top) grown in a greenhouse in indigenous soil over three consecutive years. In each of the three consecutive years seeds received irrigation treatments equivalent to 5, 10, 15, 20, 25, 30 and 40

Germination tests under controlled conditions revealed the same general pattern of spikelet position-dependent germination as the soil seed bank field experiments. Germination of florets was sequential, with the 1^st^ floret germinating almost exclusively in the first year, and the 2^nd^ floret germinating predominantly in the second year (Fig. 4). In these two florets, germination fraction in the third year, under different amount of water supplied, was zero for the 1^st^ floret and ranged 0.00 – 0.02 for the 2^nd^ floret. The 3^rd^ floret germination fraction was close to zero in the first year, highest in the second year and still high in the third year under low but not high amount of water supplied (Fig. 4). Although the amount of water affected percentage of germinated seeds, the sequential spikelet position-dependent germination was observed under the whole range of precipitation applied.

A range of supplied water amount imitated the first rainy event of different intensity. There was no seed germination under 5 mm or rainfall and germination fraction was progressively increasing from 10 mm of rainfall on for both, 1^st^ and 2^nd^ floret in the first year of experiment (Fig. 4). In the second and third years of experiment, however, germination fraction was non-zero for both floret types even under 5 mm of rainfall. For the 3^rd^ floret, immediate (i.e. in the first year of experiment) effect of rainfall amount on germination fraction was negligible, but became important in the second and third year of the experiment (Fig. 4).

Germination fraction of the 1^st^ floret of the D origin was lower than that of the M origin in the first year under 10 and 15 mm of rainfall (χ^2^_1_ = 44.1 and 8.6, p<0.001 and p<0.01, respectively), and was higher than that of the M origin in the second year for plants which received 5 and 10 mm of rainfall (χ^2^_1_ = 26.0 and 4.1, p<0.001 and p<0.05, respectively).

Germination fraction of the 2^nd^ floret of the D origin was lower than that of the M origin in the first year under all amounts of rainfall except for the smallest (5 mm) and the largest amount (40 mm) (χ^2^_1_ = 12.8, 140.7, 42.9, 32.6, 17.3 for 10, 15, 20, 25 and 30 mm, respectively, p<0.001 for all), and was higher than that of the M origin in the second year for plants which received 10 mm and more (χ^2^_1_ = 4.7, p<0.05; χ^2^_1_ = 7.9, p<0.01; χ^2^_1_ = 6.1, p<0.05; χ^2^_1_ = 14.9, p<0.001; χ^2^_1_ = 8.4, p<0.01; χ^2^_1_ = 5.4, p<0.05; for 10, 15, 20, 25, 30 and 40 mm, respectively), and in the third year for plants that received 5 mm (χ^2^_1_ = 5.1, p<0.05).

Germination fraction of the 3^rd^ floret of the D origin was lower than that of the M origin in the first year under 20 and 25 mm of rainfall (χ^2^_1_ = 33.4 and 4.5, p<0.001 and p<0.05, respectively), and was higher than that of the M origin in the second year for plants which received 5 mm (χ^2^_1_ = 30.5, p<0.001), and in the third year for all water treatments (χ^2^_1_ = 7.2, p<0.01; χ^2^_1_  = 8.7, p<0.01; χ^2^_1_ =  31.5, p<0.001; χ^2^_1_ = 12.8, p<0.001; χ^2^_1_ = 4.1, p<0.05; χ^2^_1_ = 6.0, p<0.05; χ^2^_1_ = 4.1, p<0.05 for 5, 10, 15, 20, 25, 30 and 40 mm, respectively).

**Figure 5:**
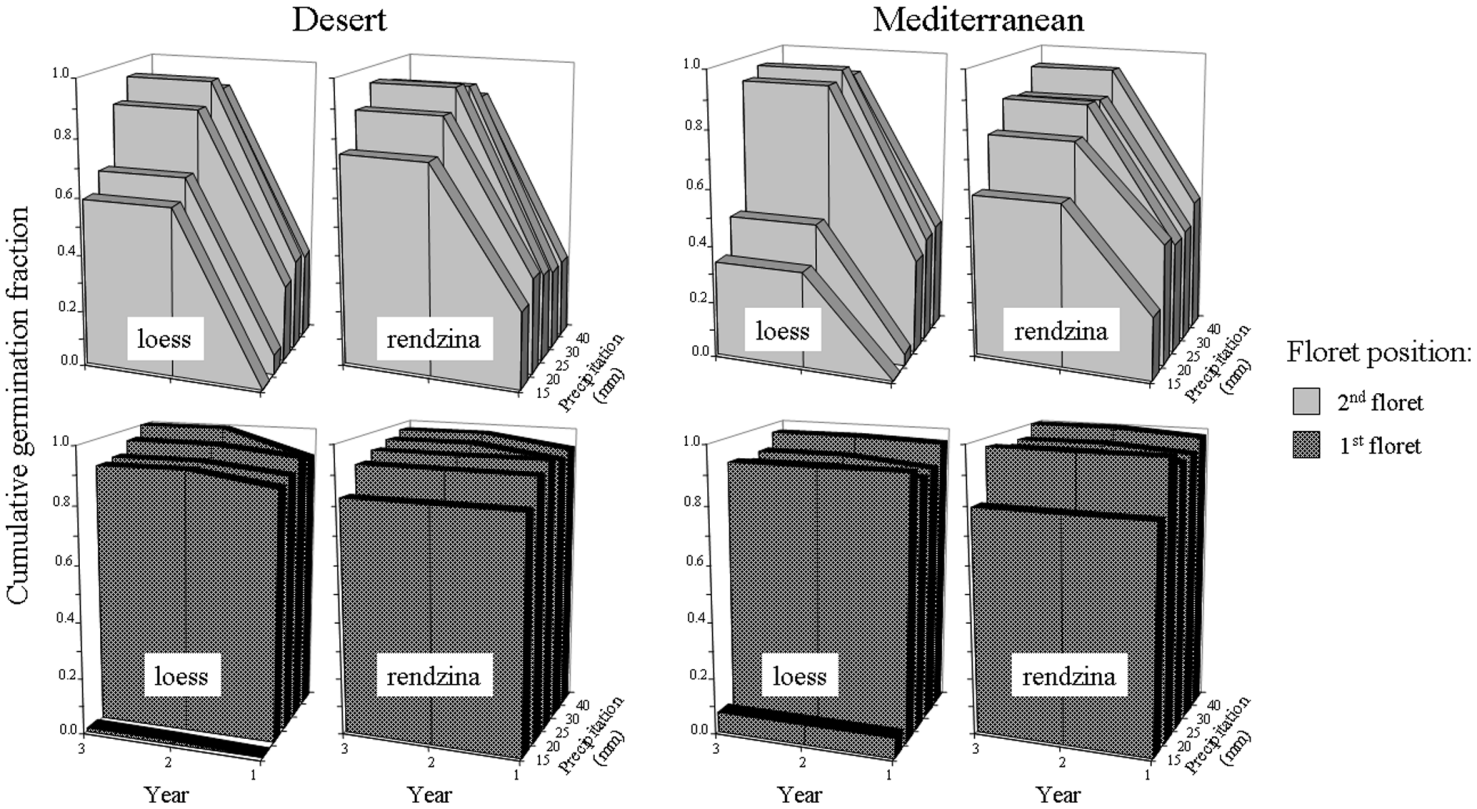
Cumulative germination fractions of the desert and Mediterranean florets of different position in a spikelet (1^st^ and 2^nd^ position in a spikelet from the bottom to the top) grown in a greenhouse in two soil types over three consecutive years. In each of the three consecutive years seeds received five irrigation treatments equivalent of 15, 20, 25, 30 and 40

The soil type had an effect on germination of the 1^st^ and 2^nd^ florets with higher percentage of germinated seeds in rendzina than in loess, especially under small amount of rainfall (Fig. 5). This effect was evident for the 1^st^ floret in the first year and for the 2^nd^ floret in the second year (Fig. 5). However, soil type did not affect either the general germination pattern of florets of different position in a spikelet or the reported above differences in germination fractions between the desert and Mediterranean origins (Fig. 5). Although germination fraction of the 1^st^ and 2^nd^ floret of the D origin did not differ from the M origin in the first year, in the second year the germination fraction of the 1^st^ floret of the D origin was exceeding that of the M origin in loess (Z = 2.0, p<0.05, Wilcoxon matched pairs test) and the germination fraction of the 2^nd^ floret of the D origin was exceeding that of the M origin in both soil types (Z =  2.0, p<0.05, Wilcoxon matched pairs test).

## Discussion

Previous studies of germination in grass species with seed heteromorphism, with a notable exception [11], were conducted either under artificial conditions or too short time frame [12]–[14]. These studies either used filter paper as a substrate instead of a native soil type, or estimated seed germination in growth chamber instead of under natural conditions, or conducted germination tests during single growing season. This is the first study where germination of seeds having different position in a spikelet is rigorously tested over time under both field and controlled conditions.

The results of both, germination tests under controlled conditions and soil seed bank field experiments, revealed the same pattern of sequential germination of the 1^st^, 2^nd^ and 3^rd^ florets with the 1^st^ floret germinating almost exclusively in the first year, the 2^nd^ floret germinating predominantly in the second year, and the 3^rd^ floret germinating almost exclusively in the second and third year. Germination is triggered by the rainy event exceeding 10 mm of rainfall. Further increase in precipitation has a positive effect on germination fraction but does not change the general pattern of floret germination. This pattern of germination is not affected by population origin or soil type.

To understand how dormancy-related seed heteromorphism evolved in *A. sterilis*, I compared the pattern of seed germination in two populations of contrasting predictability of rainfall.

Desert environments (in contrast to more mesic environments) are renown for occasional occurrence of years when successful germination is followed by complete reproductive failure [32]–[35]; [19]. This is why climate unpredictability (specifically, predictability of amount and timing of rainfall) is the major difference between the two studied environments, and seed dormancy is a known adaptation to inter-seasonal variation in precipitation. In the present study, soil seed bank experiment 1998–2000 revealed that the fraction of seeds that germinated in the second season but were dormant in a previous year, was higher at the desert vs. Mediterranean introduction site, and the D spikelets in the second season had higher germination proportion at both sites than M spikelets. The soil seed bank experiment 2005–2007 allowed a more precise insight on germination pattern in *A. sterilis* by estimating germination over time for each floret within a spikelet. This experiment detected a sequential germination of the 1^st^, 2^nd^ and 3^rd^ florets, with predominant germination of the 2^nd^ and 3^rd^ florets in the second year. However, with one exception, germination fractions of the florets during three years did not differ between the sites or were higher at the M site. Only germination of the 2^nd^ floret after one year in the soil seed bank was significantly higher at the D site.

A difference between the two field germination experiments in germination fractions at the D site in the first year after seed introduction resulted from a difference in amount and distribution of rainy events in seasons 1998/1999 and 2005/2006. When conditions for germination are met (specifically, a sufficiently large first effective rain that serves as a triggering germination threshold and adequate soil moisture during the period needed for development of a seed into seedling) [36]–[37], large fraction of seeds germinate. However, when these conditions are not met, the germination fraction is very small. This was observed in a methodologically similar study on another annual grass, *Hordeum spontaneum*, where germination fraction in the first year after seed introduction remarkably differed between two seasons, 1998/1999 and 1999/2000 [19]. This difference was because of the first effective rain (10.8 vs. 15.3 mm) and not because of the total rainfall amount in the season (39.6 vs. 35.6 mm). The difference of less than 5 mm resulted in a large increase in D seed percent germination. A 15 mm threshold of autumnal rainfall to trigger germination of most annual species was also reported for California grasslands [38]. In this study, a similar difference in the first effective rain was observed between 1998/1999 and 2005/2006 seasons (10.8 vs. 14.4 mm). The actual difference in germination conditions between these two seasons was larger because in 2005/2006, the rainy event of 14.4 mm was preceded by a rain in a previous day (4.0 mm) and followed by two days of rain (6.2 and 1.2 mm), which did not happen in 1998/1999.

In the first soil seed bank experiment, the total percent germination in the first two years (which are vital for a seed fate) was highly compromised at the D site compared to the M site, suggesting better adaptation of the D population to this unfavorable environment, at a cost of performance at the more favorable M site. Although pattern of seed germination in the field over three years inferred from the two field experiments may appear to differ little between the two populations, delayed germination and existence of the soil seed bank were clearly important for the D population, as the only means of buffering against unfavorable environmental conditions such as insufficient rainfall. In the year 1999, the desert population experienced relatively rare event of precipitation not sufficient for germinated seeds to develop into reproducing adults [39].

Results of the two experiments conducted in a greenhouse showed that the sequential germination of the 1^st^, 2^nd^ and 3^rd^ florets and the differences in dormancy of the D and M seeds observed in the field are genetically determined and not affected by soil type or precipitation. Although germination fraction was increasing with amount of precipitation after a threshold for germination of 10 mm of rainfall, there always was a fraction of the 2^nd^ and 3^rd^ florets that germinated in the second and third year, and this fraction was higher for seeds of the D as compared with the M origin.

To summarize, under controlled conditions, seeds of the desert origin had stronger dormancy and more sequential germination of florets than Mediterranean ones, which is consistent with bet hedging buffering against rainfall unpredictability. However, this difference became faint under natural conditions, suggesting involvement of other environmental factors in expression of genetically-determined dormancy. Although positional seed dormancy effect in *A. sterilis* does not appear to be a specific desert adaptation against unpredictability of rainfall events, this trait does contribute to ecotypic differentiation between desert and Mediterranean populations. Demonstration of adaptive significance of this differentiation requires tracing fate of each germinated seed till maturation and seed production, which was not assessed in this study. Nevertheless, the present findings imply that most probably positional seed dormancy effect in *A. sterilis* is not a specific desert adaptation against unpredictability of rainfall events. Heteromorphism in *A. sterilis* appears to evolve initially as a by-product of developmental constraints on seed size within a spikelet with small seeds having higher dormancy because of lower success in competition for essential metabolites or growth regulators [40]. Then, when oat plants started experiencing desert environment as either result of climate changes or range expansion, the positional seed dormancy revealed its usefulness and even necessity in a new environment and became one of the traits conferring adaptation to the desert climate unpredictability.
